# Fuelling on the wing: sensory ecology of hawkmoth foraging

**DOI:** 10.1007/s00359-019-01328-2

**Published:** 2019-03-18

**Authors:** Anna Lisa Stöckl, Almut Kelber

**Affiliations:** 1 Biozentrum, University of Würzburg, Am Hubland, 97074 Würzburg, Germany; 2 Department of Biology, Lund University, Sölvegatan 35, 22362 Lund, Sweden

**Keywords:** Sphingidae, Colour vision, Olfaction, Mechanoreception, Sensory ecology

## Abstract

Hawkmoths (Lepidoptera, Sphingidae) comprise around 1500 species, most of which forage on nectar from flowers in their adult stage, usually while hovering in front of the flower. The majority of species have a nocturnal lifestyle and are important nocturnal pollinators, but some species have turned to a diurnal lifestyle. Hawkmoths use visual and olfactory cues including CO_2_ and humidity to detect and recognise rewarding flowers; they find the nectary in the flowers by means of mechanoreceptors on the proboscis and vision, evaluate it with gustatory receptors on the proboscis, and control their hovering flight position using antennal mechanoreception and vision. Here, we review what is presently known about the sensory organs and sensory-guided behaviour that control feeding behaviour of this fascinating pollinator taxon. We also suggest that more experiments on hawkmoth behaviour in natural settings are needed to fully appreciate their sensory capabilities.

## Hawkmoth diversity and ecology

**Fig. 1:**
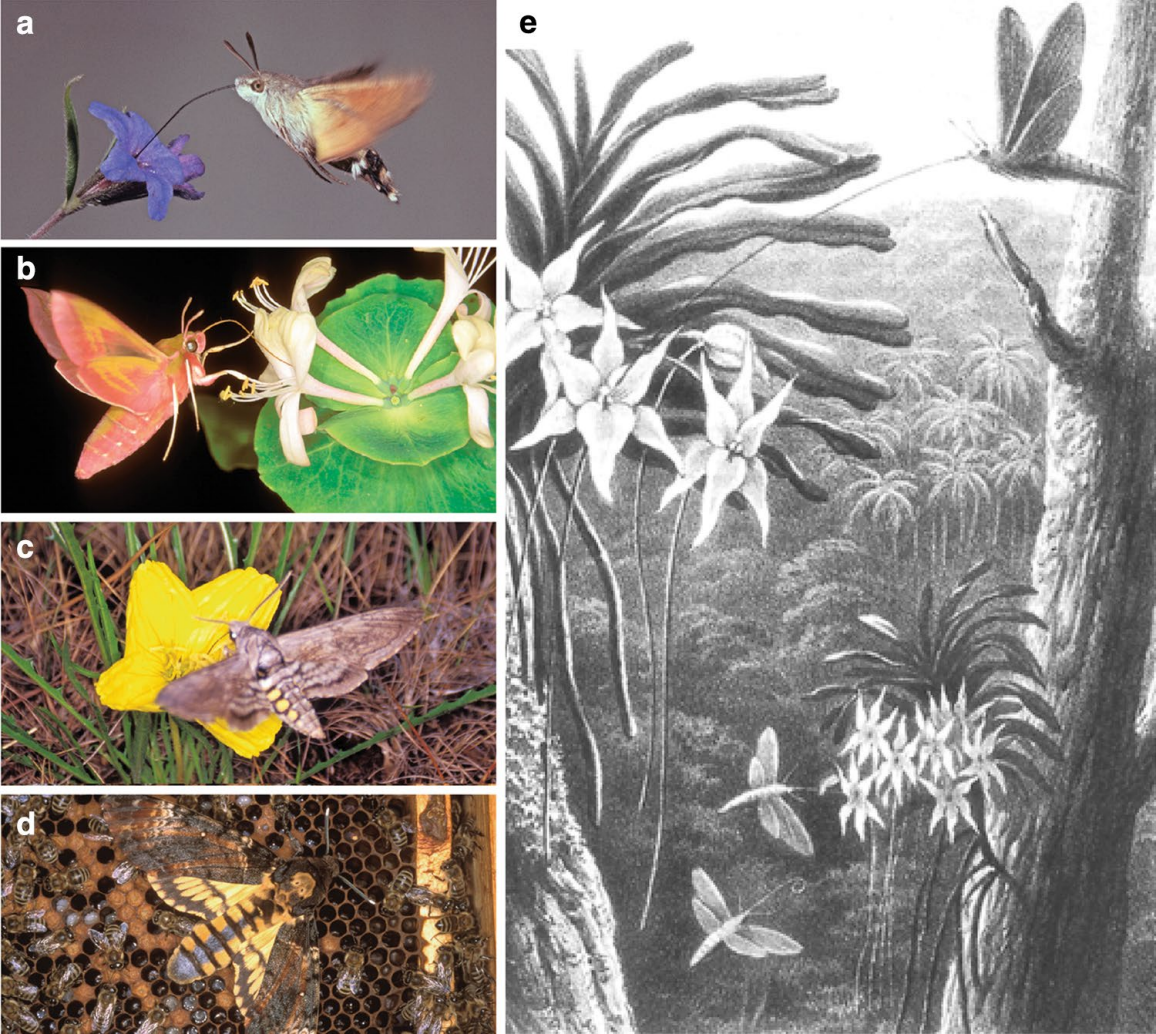
Different hawkmoth species and their food flowers. The hummingbird hawkmoth *Macroglossum stellatarum* (**a**) is one of the few day active Sphingids. It overlaps in large parts of its Eurasian habitat and many food plants with the nocturnal elephant hawkmoth *Deilephila elpenor* (**b**). Moths of the genus *Manduca*, here *M. quinquemaculata* (**c**) are popular models for olfactory and flight control research. They are distributed over most of the American continent and are nectar feeders like *M. stellatarum* and *D. elpenor*. The death’s head hawkmoth *Acherontia atropos* (**d**) is well known from popular culture. It does not feed on nectar, but extracts honey from honeycombs using its short needle-like proboscis. On the other end of the spectrum of proboscis lengths lies *Xanthopan morganii* (**e**), which has the longest proboscis amongst Sphingidae with an impressive average of 22 cm (Photos: Michael Pfaff, illustration in **e** from Wallace 1867)

Sphingidae are a well-defined family of Lepidoptera, comprising over 1450 species, mostly in the tropical, but also the temperate zones (Kawahara et al. 2009). They are characterised by streamlined bodies, narrow wings, and rapid, sustained flight. Many of them suck nectar while hovering in front of flowers (Fig. 1a–c), a highly energy-consuming flight mode which requires accurate flight control (Bartholemew and Casey 1978). Nevertheless, it is a fast and efficient feeding strategy that helps them to escape ambush predators at the flower—hawkmoths do not have to land on flowers but only contact them with their proboscis. Most famous for its long proboscis is *Xanthopan morganii praedicta* (Fig.1e), whose existence was predicted by Charles Darwin (1862) based on samples of *Angraecum sesquipedale*, an orchid in Madagascar with a 200–350 mm-long nectar spur. *Xanthopan*, with its ≈ 220 mm-long proboscis, has co-evolved with these flowers (Netz and Renner 2017) and is able to empty the nectar (Arditti et al. 2012; Johnson et al. 2017). In the neotropics, several species such as *Amphimoeca walkeri* also have 250 mm-long proboscides (Müller 1873; Johnson et al. 2017). On the opposite end of the feeding spectrum is the Death head hawkmoth *Acherontia atropos *(Fig. 1d), known from the movie *The silence of the lambs*. It intrudes into bee hives and uses its 10 mm-long, 1 mm-thick proboscis as an injection needle to pierce and suck honey from honey combs. Only species in the subfamily Smerinthinae that do not feed as adults, such as the eyed hawkmoth *Smerinthus ocellatus*, have shorter proboscides (Pittaway 1993).

Most hawkmoths, however, visit flowers and feed on nectar—in fact, they are among the most prevalent moth pollinators (Hahn and Brühl 2016). Hawkmoth-pollinated flowers often share characteristics such as white (though not ultraviolet-reflective) or yellow colour, a long nectar tube or spur, the lack of a landing zone, abundant nectar and nocturnal anthesis (van der Pijl 1961; Borges et al. 2016). They commonly share heavy-sweet odours, dominated by specific compounds. Acyclic terpene alcohols, their corresponding hydrocarbons, benzenoid alcohols and esters, and small amounts of some nitrogen compounds are typical for moth-pollinated flowers, while oxygenated sesquiterpenes seem to distinguish sphingophilous (hawkmoth-pollinated) from phalenophilous (noctuid-pollinated) flowers (Knudsen and Tollsten 1993; Miyake et al. 1998; Riffell et al. 2013). Flower species with long nectar spurs are highly specialised to be pollinated by long-tongued hawkmoths, but hawkmoths tend to be flexible and polyphagous flower visitors (Johnson et al. 2017).

Most hawkmoth species spend the day resting and are active during the twilight period and night, but a number of species within the subfamily Macroglossinae have a diurnal lifestyle, among them species of the genera *Aellopus, Amphion, Cephonodes, Hemaris*, and *Macroglossum*. The family is also diverse with respect to migration behaviour. Many species complete their life cycle and lay eggs where they enclosed from the pupae, but others migrate over long distances (Gregg et al. 1993; Pittaway 1993). For flower-visiting insects, this requires adaptation to the changing availability of different floral resources on their migration route. In addition, the combination of high wing loads, hovering flight, and long-distance migration leads to very high energy demands and, thus, the need to find rewarding flowers fast and efficiently.

In this review, we summarise what is known to date about the senses and sensory-guided strategies used by hawkmoths for finding flowers, reaching the nectar, and maintaining their position in front of the flowers while feeding. As they have been studied most thoroughly, we will focus on the nocturnal *Deilephila elpenor* (Fig. 1b), the crepuscular *Manduca sexta*, and the diurnal *Macroglossum stellatarum *(Fig. 1a), but add information on other species where available.

## Hawkmoth senses

### Eyes and vision

Within Lepidoptera, hawkmoths possess some of the largest eyes (Yagi and Koyama 1963). As typical for nocturnal insects, these are superposition compound eyes (Warrant et al. 2003). The eyes of *M. stellatarum* contain ≈ 6000 ommatidia, those of *D. elpenor* ≈ 11,500 ommatidia, and those of the large species *M. sexta* and *A. atropos* around 25,000 ommatidia (Stöckl et al. 2017b; AK unpublished data for *A. atropos*). In the dark-adapted state, a clear zone allows light from a given direction to pass through any of several hundred facets (over 3000 in *M. sexta*, but only ≈ 250 in *M. stellatarum*, Stöckl et al. 2017b), to reach the photoreceptors in a single ommatidium. This greatly improves visual sensitivity in dim light. In bright light, screening pigment migrates into this clear zone turning the superposition eyes into functional apposition eyes and adapting the visual system over several orders of magnitude of light intensity (Warrant et al. 2003; Stöckl et al. 2017b).

**Fig. 2:**
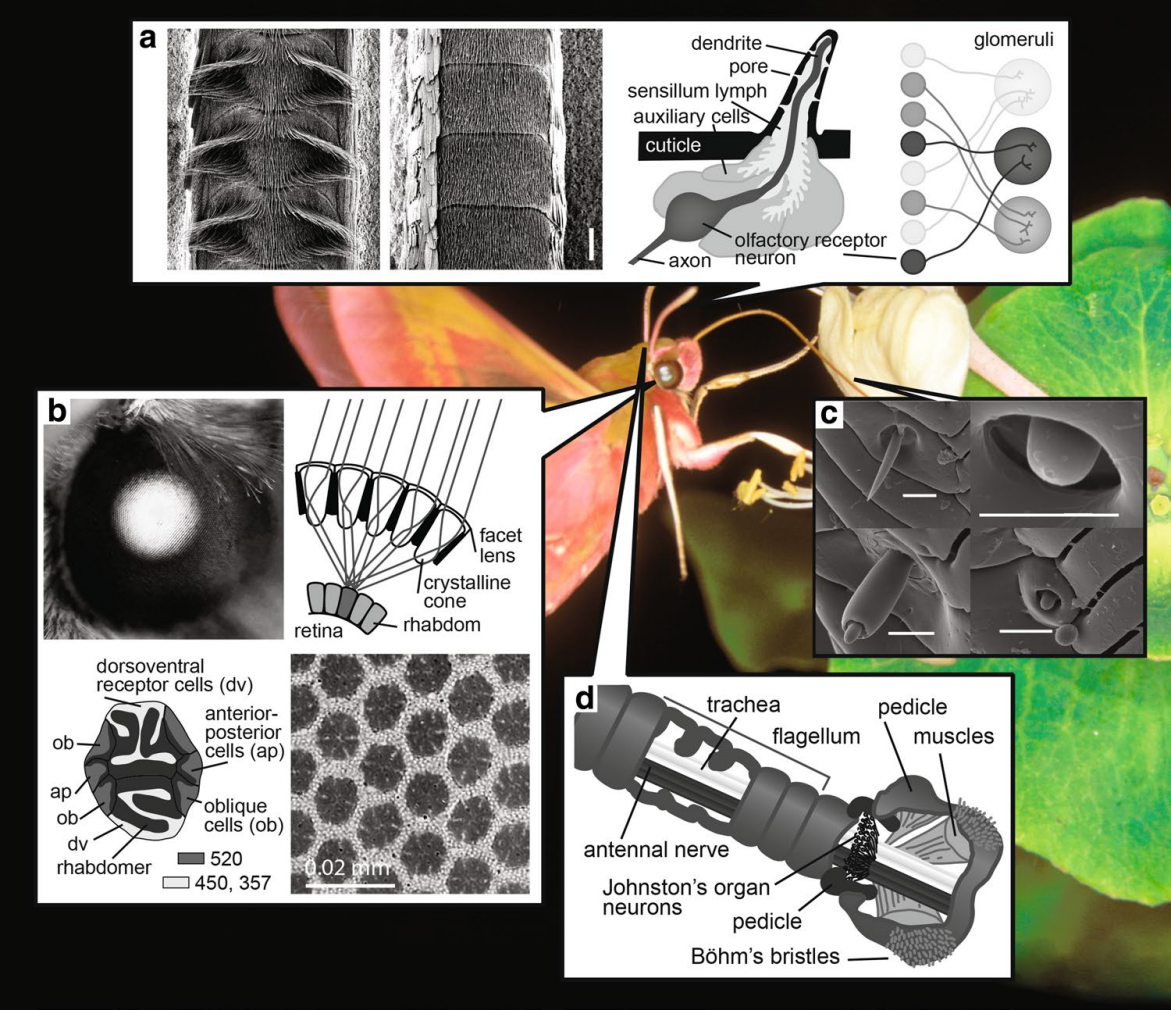
Hawkmoth senses. **a** The antennae of hawkmoths (left a male, right a female *M. stellatarum*, scale bar 100 µm) contain thousands of olfactory sensilla. Each sensillum contains dendrites of one or more olfactory receptor neurons. Axons of olfactory neurons with the same olfactory receptors project to the same glomerulus in the antennal lobes (modified after Balkenius et al. 2006; Haupt et al. 2010). **b** Hawkmoth eyes show a pseudopupil, a result of their superposition compound eyes. Light from several facets is focussed onto a single ommatidium, strongly increasing sensitivity. Each ommatidium contains 9 photoreceptor cells—2 receptors expressing UV- or blue-sensitive opsins (dv, sensitive to 357 and 450 nm in *M. sexta*), 6 receptors expressing green-sensitive opsin (ap, ob, 520 nm), and one basal cell (not shown) likely green-sensitive (White et al. 2003). Each ommatidium is surrounded by a tracheal tapetum, which reflects light through the superposition pupil and is responsible for the eye glow. **c** The hawkmoth proboscis contains gustatory and mechanosensory sensilla assessing nectar quality (top row: sensillum ampullaceum, s. chaeticum, bottom row: s. basiconicum, s. styloconicum, scale bars: 100 µm; modified from Kelber 2003). **d** The antennal base carries Johnston’s organ with mechanoreceptors involved in the control of head and body posture and Böhm’s bristles with receptors controlling antennal position (modified from Kloppenburg et al. 1997)

Hawkmoth eyes (Fig. 2b) do not only possess high visual sensitivity; they also retain high spatial acuity. While the large eyes of the nocturnal *M. sexta* and *D. elpenor* have interommatidial angles around 1°—comparable to similarly sized butterflies (Takeuchi et al. 2006), their effective spatial acuity measured as acceptance angles of the photoreceptors is 3–4 times lower (Stöckl et al. 2017b), potentially due to optical imperfections related to the large superposition pupils (as described for dung beetles by McIntyre and Caveney 1985). The diurnal *M. stellatarum*, on the other hand, retains much of the high spatial acuity that the sampling basis of their eyes provides: on average, their photoreceptors acceptance angle is 1.6°. Atypically for superposition eyes, *M. stellatarum* possesses a retinal acute zone in the frontal–ventral region of its eye and along the equator, where the optical spatial acuity approaches 1° (Warrant et al. 1999).

The temporal tuning of the green-sensitive photoreceptors of the diurnal *M. stellatarum* (Stöckl et al. 2017b) is similar to that of other diurnal Lepidoptera (e.g., *Papilio xuthus*, Kawasaki et al. 2015), with a temporal resolution limit (50% drop from maximum response) at around 50 Hz. At the same light intensities, the photoreceptors of the nocturnal hawkmoths *M. sexta* and *D. elpenor* respond distinctly slower and already reach their 50% cut-off at around 20 Hz (Stöckl et al. 2017b), similar to other nocturnal insects (Frederiksen et al. 2008).

Hawkmoths have trichromatic colour vision based on three spectral receptor types sensitive to ultraviolet, blue, and green light (see Table 1 and references therein; Kelber and Hénique 1999; Kelber et al. 2002; Telles et al. 2014). In *M. sexta*, as in other Lepidoptera, three types of ommatidia have been described, one with ultraviolet and green receptors, one with blue and green receptors, and one with all three receptor types. These ommatidial types locally build a stochastic mosaic on the eye (as suggested for another Lepidopteran species, *Papilio xuthus;* Perry et al. 2016), overlaid by a dorso-ventral gradient: blue-sensitive receptors are sparse in the dorsal eye region, whereas the ventral retina  contains mostly ommatidia with all three receptor types (Bennett et al. 1997; White et al. 2003). The spectral sensitivity, which has been studied in *M. stellatarum* and *M. sexta* in the context of feeding, qualitatively follows the sensitivity of the photoreceptors. Quantitatively, the sensitivity is between 10 and 100 times higher for blue light (of wavelengths around 440 nm) than for lights of long wavelengths (of around 520 nm), which cannot be understood from receptor properties including receptor noise alone, but indicates top-down regulation processes that control colour salience (Cutler et al. 1995; Telles et al. 2014). *M. stellatarum* is capable of discriminating lights differing in wavelength by only 1–2 nm in two narrow optimal ranges around 400 and 480 nm, and, thus, has a somewhat lower spectral resolution than the butterfly *P. xuthus*, but higher than the honeybee *Apis mellifera* (Telles et al. 2016). They can also discriminate colours, specifically spectrally similar colours, by means of intensity-related cues (Kelber 2005).

**Table 1:** Spectral sensitivity peaks (nm) of Sphingid photoreceptor types

Species	UV	Blue	Green	References
*Manduca sexta*	357	450	520	Bennett and Brown (1985)
*Deilephila elpenor*	345–350	440–450	520–525	Höglund et al. (1973), Schwemer and Paulsen (1973)
*Macroglossum stellatarum*	349	440	521	Telles et al. (2014)

Hawkmoths possess colour constancy: *M. stellatarum* and *D. elpenor* recognise the same flower colour under changing illumination spectra (Balkenius and Kelber 2004). This ability is especially important for foragers that are active under a range of lighting conditions, such as sunlight and shade, and during dawn and dusk (Johnsen et al. 2006; Kelber and Roth 2006).

The neural control centres for vision, the optic lobes and the anterior optic tubercle, have been described in several species of hawkmoths, both in their coarse anatomy (for *M. sexta*, see el Jundi et al. 2009; for *M. stellatarum* and *D. elpenor*, see; Stöckl et al. 2016), and by the physiological responses of wide-field motion-sensitive neurons (e.g., Collett 1971; O’Carroll et al. 1997; Kern 1998; Wicklein and Varjú 1999; Stöckl et al. 2017b) and looming-sensitive neurons (Wicklein and Strausfeld 2000) in the third optic neuropile, the lobula complex. The neurons that compute optic flow are crucial for flight control in insects (Borst 2014). In hawkmoths, they are sensitive to distinctly lower temporal frequencies than in diurnal butterflies and different bee or fly species (response maxima of *M. stellatarum*: 3 Hz, *Hemaris fuciformis* 2 Hz, *D. elpenor*: 1 Hz, *M. sexta*: 2 Hz, O’Carroll et al. 1996, 1997; Stöckl et al. 2017b; diurnal butterflies *Inachis io* and *Vanessa atalanta*: 10 Hz; bee-fly *Bombylius major*: 10 Hz). The maximum response decays to 50% at about 50 Hz in the butterflies, while, even in the diurnal *M. stellatarum*, this cut-off is at 12 Hz. This slow temporal tuning likely is an adaptation to the control of hovering flight (O’Carroll et al. 1996), during which the visual surrounding moves at very low speeds across the eye.

Possible functions of the internal ocelli (Warrant et al. 2003) and photosensitive cells in the adult stemmata (Lampel et al. 2005) are unknown.

### Mechanical and chemical senses on the antennae and labial palps

The antennae of hawkmoths (Fig. 2a, d) consist of three main segments, the basal scapus and pedicellus as well as the long flagella with tens of annuli. The basal two segments carry important mechanosensory organs: Böhm’s bristles, a type of sensilla chaetica, are located in three distinct fields on the scapus (with ≈ 170 bristles each in the species *Daphnis nerii*) and two fields with fewer bristles on the pedicellus (Sant and Sane 2018). Johnston’s organs are embedded within the cuticle, in the joint between scapus and pedicellus (Fig. 2d), and are sensitive to fast passive antennal movements with low amplitudes (Sant and Sane 2018). Axons of sensory cells innervating these sensilla arborize in the antennal mechanosensory and motor centre in the deutocerebrum of the moths, close to the dendritic arborisations of antennal motor neurons (Kloppenburg et al. 1997; Sant and Sane 2018).

The antennal flagellum consists of ≈ 50 annuli in the small *Macroglossum stellatarum*, ≈ 70 annuli in *Deilephila elpenor* and ≈ 90 annuli in *Manduca sexta*. Each annulus carries scales on the trailing edge and large numbers (more than 2000 in *M. sexta*) of sensilla (Fig. 2a) sensitive to plant and flower odours. According to their differently shaped cuticular structures, the sensilla are classified as sensilla trichoidea, s. basiconica, s. coeloconica, s. chaetica, and s. auricillia (Lee and Strausfeld 1990; Shields and Hildebrandt 1999a, b; Balkenius et al. 2006; Ghaninia et al. 2014). Each sensillum is innervated by one (some s. trichoidea) to five (s. coeloconicum type A) bipolar chemosensory cells, which send their axons to the antennal lobes. As in other animals, all chemosensory neurons expressing the same olfactory receptor send their axons to the same glomerulus (Haupt et al. 2010; Bisch-Knaden et al. 2018). Hawkmoth antennae are sexually dimorphic with respect to the number of sensilla: the main difference is the occurrence of two rows of long sensilla trichodea with sensory cells sensitive to the species-specific pheromones in males, but numbers of other sensilla also differ slightly between the sexes (Shields and Hildebrandt 1999a, b). In addition, a single sensillum styloconicum (also called styliform complex) innervated by one temperature-sensing and two humidity-sensing neurons (Hallberg et al. 2003; Haupt et al. 2010) is situated at the distal margin of the leading edge of each annulus (Shields and Hildebrandt 1999b; Balkenius et al. 2006).

In the antennal lobes, the number of glomeruli roughly reflects the number of olfactory receptor types; it tends to be equal or somewhat higher in females than males and differs between species (see Table 2 and references therein). Each type of odorant molecule is represented by a reproducible activity pattern in the glomeruli, leading to a functional map coding for far more odorants than the number of glomeruli (Bisch-Knaden et al. 2012, 2018).

**Table 2:** The number of glomeruli in the antennal lobes of different Sphingid species

Species (subfamily)	Number of glomeruli in females (f), males (m), unknown (u)	References
*Smerinthus ocellata* (Smerinthinae)	64–65 (u)	Bisch-Knaden et al. (2012)
*Macroglossum stellatarum* (Macroglossinae)	77 (f), 77 (m)	Stöckl et al. (2016)
*Deilephila elpenor* (Macroglossinae)	77 (f), 76 (m)	Stöckl et al. (2016)
*Agrius convolvuli* (Sphinginae)	≈ 60 (f), 58 (m)	Nirazawa et al. (2017)
*Manduca sexta* (Sphinginae)	70 (f), 68 (m)	Grosse-Wilde et al. (2011)
*Acherontia atropos* (Sphinginae)	65–68 (u)	Bisch-Knaden et al. (2012)

Additional chemoreceptors are found in the labial-pit organs on the labial palps, which also contain sensory cells sensitive to CO_2_. Their axons project to a specific labial-pit organ glomerulus in the antennal lobes (Guerenstein et al. 2004). In some hawkmoths (among these the genera *Acherontia, Hippotion*, and *Hyles*), the labial palps have also evolved ultrasound-sensitive organs allowing their owners to hear and escape echolocating bats (see below; Roeder et al. 1968, 1970; Göpfert et al. 2002).

As hawkmoths detect and orient towards flower odours while flying, the air flow over the antennae—and with it the perceived odour concentration—is modulated at the wing beat frequency. Indeed, the moths are specifically sensitive to odour stimuli pulsed with their wing beat frequency, and this coupling is mediated by neurons sending information from the flight control neurons to the antennal lobes (Chapman et al. 2018).

### Sensory organs on the proboscis

Finally, the tip of the proboscis of hawkmoths also carries olfactory receptors that sense the odour of the nectar (Haverkamp et al. 2016a). In addition, the long proboscis is covered with a large number of sensilla (Fig. 2c) containing gustatory and mechanosensory receptors allowing the moths to sense the nutrient content of the nectar which they are ingesting (Kelber 2003). As shown in other moths (Kvello et al. 2006), they project to the suboesophageal ganglion (Reiter et al. 2015), where information on tastant chemicals is represented in a spatiotemporal activity code. *M. stellatarum* prefers sucrose to fructose and fructose to glucose (Kelber 2003), and if ingesting pure sucrose solution, a concentration of 20–40% (Josens and Farina 1997, 2001). The proboscis mechanosensors relay information on surface properties of the flower during probing (Goyret 2010; Goyret and Kelber 2011), as well as on the position of the proboscis with respect to the head (discussed for butterflies by Krenn 1998).

### Sensory integration

Neurons from the antennal lobes project via several antenno-cerebral tracts to the mushroom bodies but also to other regions in the protocerebrum of the moth (Homberg et al. 1988). The mushroom bodies are the central brain structure implied in higher olfactory processing, multisensory integration and learning of insects (reviewed by Heisenberg 2003). In *M. sexta*, experiments using calcium-sensitive optical imaging have shown that the neural activity in the mushroom bodies depends not only on olfactory but also visual stimulation, in a complex way depending on the colour of the visual stimulus (Balkenius et al. 2009), the identity, and the concentration of the odour (Balkenius and Balkenius 2016) and learning processes (Balkenius and Hansson 2012).

## Finding flowers

**Fig. 3:**
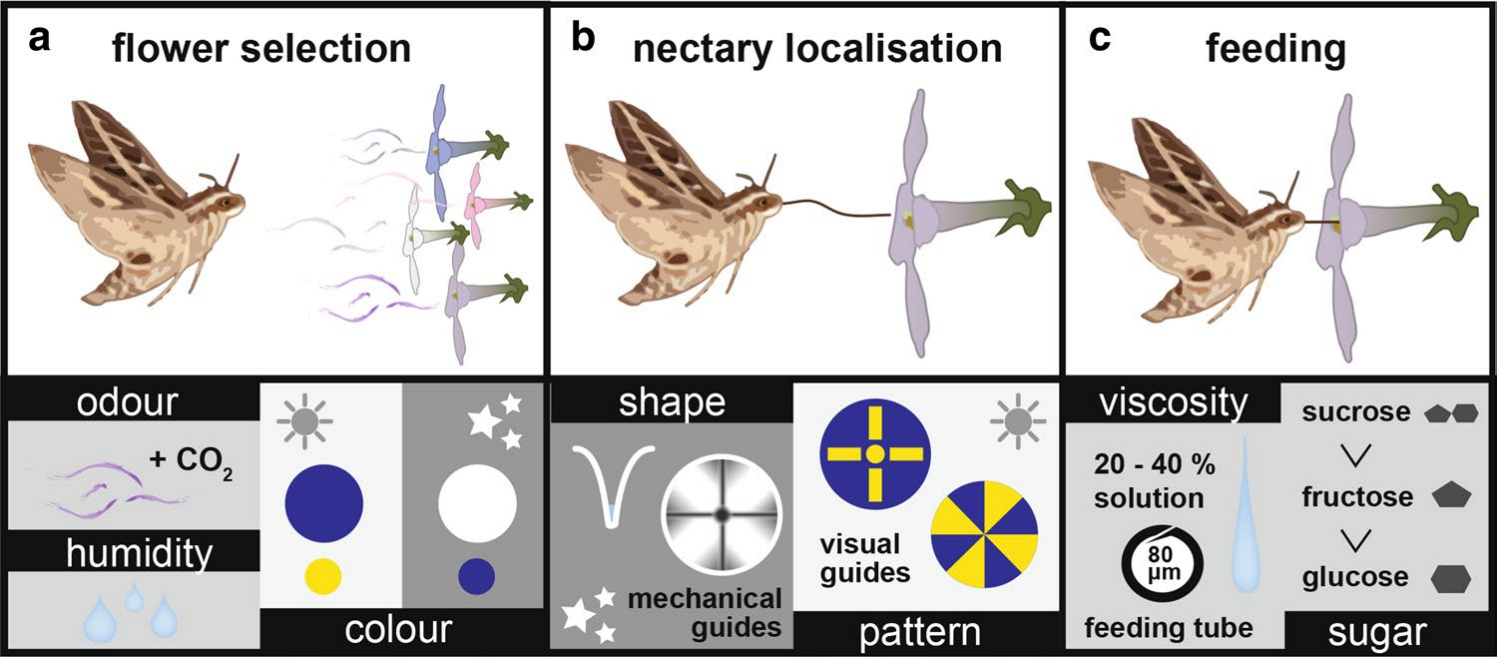
Sensory cues of hawkmoth flower selection. Hawkmoths use different sensory cues to select and evaluate flowers at different stages of approach and feeding. **a** Attraction and selection of flowers from a distance (several metres) occurs via olfactory cues, including CO_2_ sensing, as well as air humidity. Hawkmoths are also attracted by the colour of the flower. Naïve moths prefer blue (and to a lesser extend yellow) flowers at bright light intensities, and white flowers (to a lesser extend blue) at nocturnal intensities. These preferences can change with experience. **b** As moths get close to the flower, visual patterns help diurnal species to guide the proboscis to the entrance of the nectary. Nocturnal moths rely more strongly on mechanosensory cues provided by the shape and mechanical guides of the flower. **c** When moths have inserted their proboscis into the nectary, they evaluate the nectar for its viscosity and for sugars and secondary metabolites

Hawkmoths use a diversity of sensory cues to find rewarding flowers including scents, CO_2_ concentration, humidity, colour, size, shape, pattern, orientation, and spatial location. Depending on the distance to the flowers (Raguso 2008) and their ecology, different species give different weights to different sensory modalities and cues (Fig. 3a). Being solitary insects, finding the first nectar source requires guidance by innate preferences for flower features that typically promise a reward. Hawkmoths with a long life-span and specifically migrating species also strongly depend on their ability to learn specific odours, colours, and other features of rewarding flower species.

### Odour cues

Many flowers, specifically those with nocturnal anthesis (Borges et al. 2016), announce the availability of nectar by emitting strong scents, mostly from the petals and the nectar (e.g. Kessler and Baldwin 2006). Flower odours have direct and indirect functions, both as distance attractants, feeding cues and as synergists with other sensory cues (Raguso and Willis 2002; Raguso 2008). A nocturnal hawkmoth such as *D. elpenor* predominantly orients towards typical flower scents both when searching for the first flower (Balkenius et al. 2006) and after collecting experience with rewarding flowers (Stöckl et al. 2016). The preferences of naive moths for specific odours can be highly adaptive, leading the hawkmoth to flowers with high rewards. Haverkamp et al. (2016b), for instance, showed that *M. sexta*, given the choice between the odours of several species of tobacco, prefer the odour of *Nicotiana alata*, which happens to have a flower tube of the optimal length for the moths to extract nectar and gain most energy. In the same species, the behaviourally demonstrated innate preference is mirrored by the mean firing rates and patterns of synchronously firing in neurons in the antennal lobes (Riffell et al. 2009). This is probably facilitated by high sensitivity to specific odorants such as benzenoids and oxygenated monoterpenes that are typical for nocturnal flowers (Riffell et al. 2008).

In addition to having such innate preferences, hawkmoths can flexibly learn and react to other odour signatures allowing them to easily adapt to changing flower resources (Balkenius et al. 2006; Riffell et al. 2008; Stöckl et al. 2016). Specifically when hawkmoths use the same plant as a nectar source and an oviposition substrate, olfactory preferences for nectar sources can be sexually dimorphic. Females of *M. sexta* spend only half of a visit to a *Datura wrighty* flower feeding, and half ovipositing, while males can invest in feeding only (Alarcón et al. 2010). Similar relationships may be expected for *Hyles lineata* that feeds and oviposits on *Oenothera* (e.g., Mock and Ohlenbusch 1981).

Hawkmoths also make use of additional chemical cues. *M. sexta* can use elevated CO_2_ concentrations and humidity as a predictors for highly rewarding flowers from a distance of up to 3 m, as shown in wind-tunnel assays (Goyret et al. 2008a; von Arx et al. 2012).

### Visual cues

Typical hawkmoth-pollinated flowers often have a bright colour, indicating that hawkmoths use vision as an additional sense to find nectar sources. Unlike nocturnal hawkmoths, the diurnal species *M. stellatarum* puts more weight on visual cues than odours (Balkenius et al. 2006; Stöckl et al. 2016), and for the naive animal visiting its first flower, floral colour is given the highest weight, followed by size and pattern (Kelber 1997). It may be interesting to note, however, that another diurnal hawkmoth species, *Amphion floridensis*, which feeds on sap and rotten fruit, gives a much lower weight to flower colour (AK, personal observation).

Similar to other flower-visiting insects, *M. stellatarum* has a strong innate preference for blue (440 nm) and a weaker preference for yellow (or wavelengths of 540 nm). This mirrors their spectral sensitivity (see above; Telles et al. 2014), indicating that the mechanism underlying their innate preferences likely involves top–down control of spectral sensitivity, which may be context-dependent. In addition, these colour preferences are flexible and depend on background and illumination colours, such that the preference for yellow can be stronger in bluish illumination or on a blue background (Kelber 1997). Under crepuscular illumination conditions, even *M. sexta*—known to visit white nocturnal flowers in its natural habitat—shows an innate preference for blue, compared to other colours (Cutler et al. 1995) and also to white (Goyret et al. 2008b). Again, this preference depends on both illumination and background (Kuenzinger et al. 2019).

Just as with odours, all hawkmoth species tested so far change their preference for flower colours with experience. *M. stellatarum* remembers a colour associated with a nectar reward after a single learning event (Kelber and Hénique 1999), *D. elpenor, H. lineata*, and *Hyles livornica* can learn flower colours in dim starlight conditions (Kelber et al. 2002), and *M. sexta* can learn to prefer white flowers under conditions in which it innately prefers blue (Goyret et al. 2008b). Interestingly, while most hawkmoth species hibernate as pupae, *M. stellatarum* hibernate as imagines, allowing for tests of their long-term memory. Colour preferences learned prior to hibernation can fade away already after 3 weeks, and the animals return to their innate preferences (Kelber 2010), indicating that forgetting may be equally important as fast learning in a world of changing resources.

Additional visual cues used by hawkmoths include the shape, size, and patterns of flowers. For *M. stellatarum*, the “optimal” flower is blue, has diameter of around 30–40 mm and a contrasting radial pattern (Kelber 1997, 2002). Hawkmoths can also remember the spatial location of a rewarding flower (Balkenius et al. 2004) and finally, they select for the orientation of a flower. Some flowers change the orientation of the corolla during the course of the day, and with it the ease of access to the nectary changes. The crepuscular *H. lineata* and several African hawkmoth species prefer upward-facing flowers, which provide a better access to the nectary, to downward-facing corollas (Fulton and Hodges 1999; Campbell et al. 2016). Interestingly, the nocturnal *M. sexta* approaches both upward- and downward-facing flowers with the same probability (Haverkamp et al. 2019), suggesting that, similar to other visual cues, flower orientation is less important than olfaction for their flower choice.

### Sensory integration

As pointed out above, hawkmoths may give higher weight to olfactory or visual flower cues, depending on their ecology and the context. Both sensory modalities may also play more or less dominant roles at different stages in the approach to the flowers. Naive *M. sexta*, for instance, seem to be attracted to flower arrays from a distance by scent (Raguso and Willis 2002, 2005), but use visual cues at close distance and only extend the proboscis for feeding when they can see the flower (Balkenius and Dacke 2010). They are flexible and show a more prominent preference for scented vs scentless flowers when visual cues are less reliable (Goyret et al. 2009), as is the case in dimmer light (Goyret and Yuan 2015). *M. sexta, M. stellatarum* and *D. elpenor*, seem to react to flower colour and odour independently, and do not perceive the scented and coloured flower as a unique fused target (Balkenius and Dacke 2013; Stöckl et al. 2016). In addition to floral scents, hawkmoths also base their flower choice on humidity (von Arx et al. 2012) and CO_2_ concentrations close to the flower (Goyret et al. 2008a), both of which are good predictors of the presence of nectar.

## Finding and evaluating the nectar

When hawkmoths have chosen a promising flower and approach it with extended proboscis, the next challenge is finding the entrance to the nectar reservoir (Fig. 3b). To find this entrance, most species of hawkmoths probe the flower with the tip of their proboscis while hovering in front of the flower. It is quite impressive to see how accurately a hawkmoth can control its proboscis − 25 mm long in *M. stellatarum* and *D. elpenor*, but 100 mm in *M. sexta* or *A. convolvuli* and over 200 mm in *X. morgani*—to retrieve and empty the reward fast and efficiently. Mechanical senses and vision are involved in controlling this task. In a next step, they have to evaluate the quality of the nectar by means of taste receptors (Fig. 3c).

### Visual nectar guides

Most flowers are not uniformly coloured. As already observed by Sprengel (1793), many flower patterns serve as nectar guides. They allow *M. stellatarum* to find and empty the nectar of several hundred small flowers, for instance of *Gentiana bavarica*, within a few minutes (Müller 1881). This hawkmoth species switches colour preference during a single flower visit: a naive moth, after being attracted to the blue overall colour of flowers (see above), preferentially probes yellow areas with the proboscis, using both chromatic and achromatic contrast (Goyret and Kelber 2011, 2012). For *M. stellatarum*, visual guides have higher priority and can override mechanical cues (Goyret and Kelber 2011). The long-tongued *M. sexta*, by contrast, can only make partial use of  visual cues (Goyret and Raguso 2006), most likely because it can barely visually resolve its own proboscis tip with the eyes.

### Flower shape and mechanical nectar guides

In contrast to many flower models used in experiments, real flowers are three-dimensional structures, and the funnel-shaped flowers of many hawkmoth-pollinated flower species such as *Datura wrightii* or *Oenothera sp*. have evolved to allow easy access of their pollinators to the nectar reward. Even sharply curved trumped-shaped flowers function very well for guiding *M. sexta* to the nectar (Campos et al. 2015). Hawkmoths can also use grooves leading from the edge to the centre of the flower to guide the proboscis to the nectary (Goyret and Raguso 2006; Goyret and Kelber 2011). Inexperienced animals find the nectary faster in artificial flower models with such mechanical guides than in flat flowers. On flat flowers, the search time depends on the area that needs to be probed: *M. sexta* found the nectary faster when searching on smaller than larger circular flowers, and on flowers with separated petals, compared to circular flowers with the same diameter (Goyret and Raguso 2006).

### Evaluating nectar quality

Almost all adult hawkmoths feed exclusively on nectar, which contains sugars and only small amounts of amino acids, fatty acids and minerals. Long-lived hawkmoths use ingested nectar carbohydrates and fatty acids as fuel for flight (O’Brien 1999) and allocate carbohydrates and amino acids in eggs and flight muscles (O’Brien et al. 2000; von Arx et al. 2013; Levin et al. 2017). Thus, hawkmoths should be able to sense and base flower choice on the quality of nectar. Many pollinating insects use tarsal taste sensilla for this task, but, since hawkmoths are feeding on the wing, they can only use the sensilla on the proboscis to evaluate the concentration and ingredients of nectar. High sugar concentrations go along with high viscosity which hinders fast uptake of nectar with the long proboscides of most hawkmoths. This, together with the need to keep a sufficient water balance, is likely the reason for their preference for sugar concentrations between 20 and 40% (Josens and Farina 1997; Contreras et al. 2013). The preferences of hawkmoths for sucrose over fructose over glucose have been behaviourally established (Kelber 2003; Reiter et al. 2015), and their evaluation of the scent of nectar is undisputed (see above). While taste receptors on the proboscis of *M. sexta* have also been shown to sense secondary metabolites such as benzyl acetone (Haverkamp et al. 2016a, 2018), nicotine (Kessler and Baldwin 2006), and other components (Reiter et al. 2015; reviewed by; Stevenson et al. 2017), it is—to our knowledge—unknown how exactly they sense the presence of other non-volatile constituents such as amino acids and fatty acids.

## Position control during hovering

**Fig. 4:**
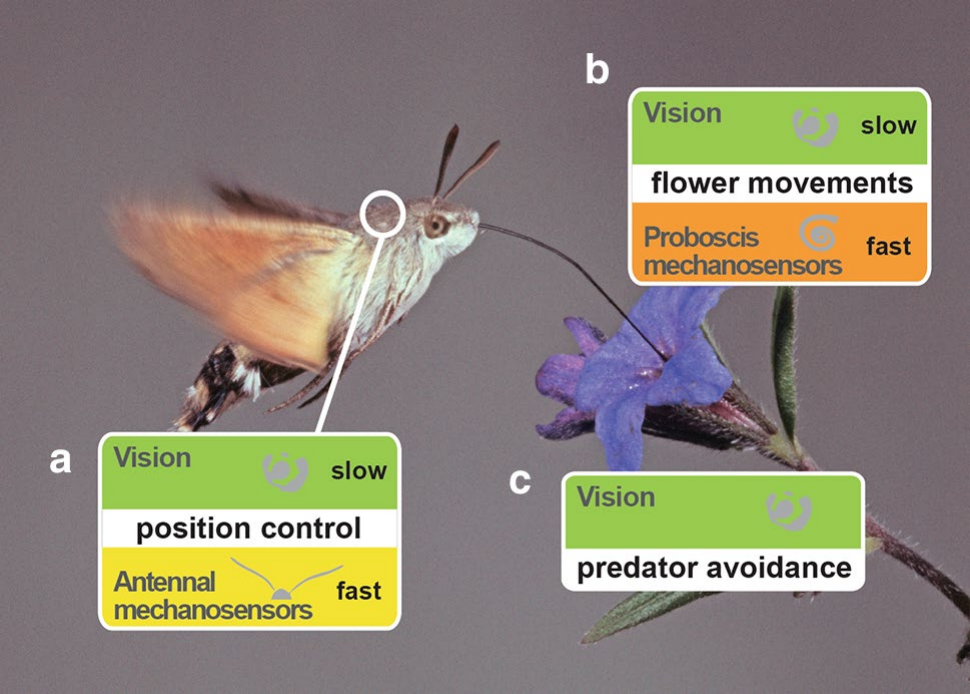
Sensory modalities controlling flight at the flower. To control their position at the flower and counteract disturbances (**a**) caused, for instance, by wind, hawkmoths use vision, and antennal mechanosensation. These senses operate in different temporal frequency ranges, with vision operating at low and mechanosensory input at high frequencies. Hawkmoths can also track the position of the flower (**b**) using vision and mechanosensory feedback from the proboscis. While hovering, hawkmoths visually sense aerial predators (**c**). Whether hawkmoths use sensory mechanisms other than vision to avoid predators at flowers is unknown, only a few species have evolved ears sensitive to bat sound

As hawkmoths do not land on the flowers, but hover in front of them, they have to maintain a stable body and flight position to keep their proboscis in the nectary  during feeding. Since hovering is an inherently unstable mode of flight, it requires continuous sensory feedback to maintain a set body position (Zhang and Sun 2011; Liang and Sun 2013) and is particularly vulnerable to perturbations, for example by wind gusts. Hawkmoths can sense positional perturbations using both vision and mechanosensation and correct for them (Fig. 4).

### Visual control of position

*M. stellatarum* responds both to wide-field translational and rotational optic flow (Farina et al. 1995; Kern and Varjú 1998) to correct for forward and backward displacements, as well as rotations relative to the nectary of the flower. Interestingly, these hawkmoths are most sensitive to the two motion components in different parts of their eyes: translational optic flow elicits the strongest responses in their frontal visual field, and rotational optic flow in the lateral visual field (Kern and Varjú 1998). This functional regionalization is well adapted to the visual scene that hawkmoths experience during feeding, when the flower takes up a large part of the frontal visual field, while the surrounding landscape is mostly visible in the lateral and posterior visual field. The visual position control is likely supported by the wide-field motion vision system, since the temporal dynamic of the compensatory movements show similar temporal properties as the wide-field motion-sensitive neurons in the lobula complex and in descending tracts (Wicklein and Varjú 1999; Kern 1998; Stöckl et al. 2017b). In tethered-flying *M. sexta*, optic flow in the dorso-lateral region has been found to be critical for flight control (Copley et al. 2018), and while the spatial resolution and contrast sensitivity decreases in dim light, the preferred temporal frequency remains around 4.5 Hz in all the light intensities (Parthasarathy and Willis 2018).

### Mechanosensory control of position

In addition to visual input, hawkmoths can use mechanosensory information to control for perturbations of their body position in flight. This is important because mechanosensory receptors can respond much faster (Yarger and Fox 2016), while the visual system is strongly limited in response speed and further slows down in dim light (Theobald et al. 2010; Stöckl et al. 2017a, b)—a challenge especially for the family of hawkmoths with predominantly crepuscular and nocturnal members. While all Lepidopterans lack halteres, the prominent gyroscopic organs of flies (Fraenkel and Pringle 1938; Pringle 1948; Nalbach 1994) which provide important sensory input to stabilise flight after perturbations (Ristroph et al. 2010), hawkmoths have been shown to use their antennae for a similar purpose. *M. sexta* is severely impaired in its ability to maintain any stable flight position without their antennal flagella (Sane et al. 2007). We recently showed that *M. stellatarum* can still fly without flagella, which allowed us to investigate the contribution of this mechanosensory system to flight control in more detail (Dahake et al. 2018). Like halteres of flies, the antennae of moths seem to be especially important for positional control during fast manoeuvres, while slower ones (below 2 Hz) can be controlled successfully by vision alone (Yarger and Fox 2016; Dahake et al. 2018). This divergence of vision and mechanosensation into two parallel channels with different dynamic ranges is also found in Dipteran flight control (Mureli and Fox 2015).

## Flower tracking

A feeding hawkmoth does not only have to worry about its own position in the air, but also about that of the flower, which might be swaying in the wind. A range of typical hawkmoth flowers shows distinct oscillatory movements even at low wind speeds, which are stronger in the horizontal than the vertical plane (Farina et al. 1994; Sponberg et al. 2015) and increase with wind speed (Farina et al. 1994), up to several centimetres of amplitude. Interestingly, the flowers of a range of different plant species have very similar frequency spectra of oscillatory movements: most of their power is concentrated below 1 Hz, and none of the measured species show any power above 5 Hz (Sponberg et al. 2015). Both diurnal and nocturnal hawkmoths readily track flower movements within this frequency range to stay aligned with the nectary and not loose contact with their proboscis. They can track front-to-back (Farina et al. 1994), up-and-down (Sprayberry and Daniel 2007), as well as sideways movements of the flower (Sponberg et al. 2015; Stöckl et al. 2017a). The intake of nectar is lower when they are tracking moving flowers, compared to feeding at stationary ones. However, the energy expended to track the flowers is minimal compared to the energy intake from the nectar (Sprayberry and Daniel 2007). Surprisingly, hawkmoths track flowers providing nectar with high sugar content more precisely than flower presenting low sugar concentrations (Farina and Josens 1994) suggesting that flower tracking is not an entirely hard-wired behaviour, but that the “effort” put into it is controlled by contextual information.

### Visual flower tracking

By decoupling the visual and mechanosensory cues of flower movement, it has clearly been demonstrated that flower tracking in hawkmoths has a distinct visual component, both in controlling the distance to the flower (Farina et al. 1994), as well as its horizontal position (Roth et al. 2016). The importance of vision is also reflected by the fact that flower tracking at higher temporal frequencies becomes less precise as light intensities decrease (Sponberg et al. 2015; Stöckl et al. 2017a), a consequence of the visual system (both photoreceptors and wide-field motion neurons) slowing down at lower light intensities (Stöckl et al. 2017b). It is not entirely clear which visual pathway is responsible for flower tracking, but experiments by Farina et al. (1994) on *M. stellatarum* give some indications: when controlling their distance to projected flowers simulating back-to-front movements, hawkmoths seem to neither use stereopsis cues nor motion parallax information, but, instead, respond to the apparent speed, at which the contour lines of the flower pattern move (Farina et al. 1994). This hypothesis is supported by the finding that the dynamic properties of their flower tracking responses show the bandpass characteristics expected from a pathway based on correlation-type motion detectors (Farina et al. 1994). If this also holds for sideways flower movement is not clear, but the frequency range at which hawkmoths track the flower movements, is definitely within the dynamic range of their wide-field motion-sensitive neurons (Stöckl et al. 2017a).

### Mechanosensory flower tracking

Hawkmoths also use mechanosensory cues from their proboscis (Roth et al. 2016), which are linearly integrated with the visual information, to follow flower movements. However, the weighting between mechanosensory and visual information during flower tracking seems to differ between species. In *M. sexta*, the weight to visual cues is distinctly lower than to mechanosensory cues, at flower movement frequencies below 2 Hz. By contrast, in *M. stellatarum*, the strength of visual tracking is only reduced by 6% when in conflict with mechanosensory tracking, suggesting that this diurnal species relies more strongly on vision for flower tracking (Farina et al. 1994).

This parallels similar differences in the weight given to different sensory modalities in the context of flower selection and nectar finding, where the diurnal species relies stronger on the visual sense, while nocturnal hawkmoths rely stronger on alternative sensory input, as their visual sense is compromised by the low-light intensities. However, since this comparison currently is only based on two species from different subfamilies, it remains speculative.

## Predator avoidance at the flower

Losing contact with the nectary is not the only danger hawkmoths face during feeding. They might also be an easier target when hovering close to flowers on which ambush predators might be waiting. Moreover, retaining a relatively stable position in the air might make them easy prey also for airborne predators. However, little quantitative data are available on which animals predate on hawkmoths. There are suggestions that hawkmoths are predated by ambush predators on flowers, such as praying mantis or spiders (Delf and Harris 1964; Wasserthal 1993), while other authors deem this less likely, especially for large hawkmoths species, and suggest that their main predation pressure is from airborne predators such as birds and bats (Nilsson 1998a, b). To avoid bat predation, hearing organs have evolved at least twice independently in Choerocampini (studied in *H. lineata* (former *Celerio lineata*, Roeder et al. 1968, 1970; and *Hippotion celerio*; Göpfert et al. 2002) and in Acherontiini (studied in *A. atropos* and *Panogena lingens*, Göpfert et al. 2002). Different structures of the labial palp have been recruited to function as tympana in these two sub-tribes, making the moths sensitive to ultrasound (Göpfert et al. 2002; Roeder et al. 1970). In addition, hawkmoths in the subtribe Choerocampina can also produce sounds in the ultrasound spectrum (around 55 kHz) when stimulated by bat sounds (studied in *Cechenena lineosa, Theretra boisduvalii*, and *Theretra nessus*, Barber and Kawahara 2013). Most hawkmoths, however, are likely deaf.

It is an interesting question to which degree hover-feeding itself is an adaptation to avoid predators, as hawkmoths keep a safe distance to ambush predators on the flower and can react much faster to attacks from the air or the flower, as they do not have to take off to initiate an escape manoeuvre. In terms of predator-avoidance behaviours, *M. sexta* perform evasive flight manoeuvres upon presentation of looming stimuli from above (Cheng et al. 2011), and in their lobula complex, looming-sensitive neurons have been described (Wicklein and Strausfeld 2000). However, it is not entirely clear whether the detection and reaction to looming stimuli serves the avoidance of predators or that of obstacles in flight, or both. It has also been suggested that swing-hovering, which is observed especially when long-tongued hawkmoths feed from flowers with short corolla, is a predator-avoidance strategy (Wasserthal 1993). While some authors question this hypothesis (Nilsson 1998a, b), recent work might support it (Haverkamp et al. 2016b). A clearer understanding of the stimuli that trigger this behaviour and functional investigations asking whether it actually detracts predators are required to understand whether swing-hovering is, indeed, an adaptive predator-avoidance strategy.

## Summary and outlook

As we have summarised here, a great wealth of studies exists on the contribution of individual sensory modalities to hawkmoth foraging, ranging from flower selection, both at a distance and in close proximity, to judging the quality of the nectar, and controlling flight while hovering in front of the flower. Some work has also shed light onto the question how the different sensory modalities interact for foraging decisions, and how the weights of these modalities are correlated with the sensory ecology of different species. Most of the described studies, however, have investigated hawkmoths in laboratory settings, with clearly defined artificial stimuli.

The next step to a complete understanding of the complexity of hawkmoth foraging will, therefore, have to be studies of foraging decisions and foraging behaviour in a more natural environment, and with more complex stimuli for each single modality. For example, hawkmoths in their natural environment are not only confronted with a single target odour, but perceive a complex bouquet of odours from multiple sources, among which they select those that are relevant for flower foraging. Riffell et al. (2014) demonstrated both behaviourally and physiologically that such background odours can temporarily reduce the ability of *M. sexta* to track the odour of their target flower.

Moreover, in natural environments, multiple sensory modalities are active at the same time. While a moth tries to discriminate an odour source from the background, visual cues might contribute to a more robust representation of the target flower than odour cues alone and, thus, facilitate flower choice.

Another interesting question is how hawkmoths stabilise their hovering flight at the flower in a dynamic environment, in which not only the flower but also the background is moving. Work on tethered-flying fruit flies suggests two independent visual systems for target tracking (stripe fixation) and perception of background motion (Bahl et al. 2013). The background information is largely suppressed in the output controlling the wings when flies are tracking a frontal object, even though the background fills a much larger part of the visual field (Fox et al. 2014). However, the head movements of fruit flies exclusively follow the background motion, acting as stabilising input supporting the object tracking behaviour (Fox and Fry 2014). Whether hawkmoths use a similar strategy when tracking moving flowers in front of a dynamic background remains an interesting question to be tested.

Finally, our knowledge on predator-avoidance strategies in hawkmoths would also greatly benefit from studies of a more natural setting. First, for a given hawkmoth species, it would be important to determine the natural predators, and the hunting strategies—perching on flowers, or attacking from the air or both. These hunting techniques might greatly influence the avoidance strategies of the hawkmoths. Studies with perching predators, such as crab spiders, have been conducted on different species of bees (Dukas and Morse 2003). Such studies might help to understand how predators contribute to flower selection by hawkmoths.

Taken together, the sensory ecology of hawkmoth foraging is ready to take the step from the laboratory to the field, and from analytic examinations using reductionist stimuli to real life. These magnificent insects certainly keep more secrets that wait to be revealed.
